# Application of a machine learning model to predict the estimated primary care patient time consumption

**DOI:** 10.1038/s44401-025-00061-0

**Published:** 2026-02-02

**Authors:** Yufei Yu, Joseph Diaz, Tsung-Ting Kuo, Alson Mo, Zach Pope, Hyeon Seok Hwang, Amy M. Sitapati

**Affiliations:** 1 Division of Biomedical Informatics, UC San Diego, San Diego, CA USA; 2 Department of Medicine, UC San Diego, San Diego, CA USA; 3 Department of Biomedical Informatics and Data Science, School of Medicine, Yale University, New Haven, CT USA; 4 Department of Surgery, School of Medicine, Yale University, New Haven, CT USA; 5 UC San Diego Health, San Diego, CA USA; 6 Department of Family Medicine, UC San Diego, San Diego, CA USA; 7 College of Medicine, Kyung Hee University, Seoul, Korea

**Keywords:** Computational biology and bioinformatics, Diseases, Health care, Mathematics and computing, Medical research

## Abstract

Rising healthcare costs and a shortage of primary care providers in the United States create substantial strain on the healthcare system, underscoring the need for efficient allocation of limited resources. Accurate prediction of high primary care utilization can enable proactive care planning, targeted interventions, and workload optimization. We developed and evaluated the Friedman Score, a machine learning–based model that predicts estimated yearly primary care utilization categories, low use (0–85th percentile), High Use (>85–95th percentile), and very High Use (>95th percentile), using structured electronic health record data from UCSD Health primary care patients in 2022–2023. Features included age, chronic disease diagnoses, medication history, and acute care patterns. XGBoost was selected as the primary modeling approach, and its results were benchmarked against five other machine learning algorithms. Across both years, XGBoost consistently demonstrated high discriminative ability (AUC 0.78–0.89 in 2022; 0.81–0.89 in 2023) and robust calibration. SHAP analysis identified medication usage, age, and chronic disease burden, particularly depression, as the most influential predictors. The Friedman Score offers a robust, interpretable tool for identifying high-utilization patients, providing actionable insights to guide proactive, data-driven primary care delivery.

## Introduction

Healthcare costs in the United States continue to rise, reaching $4.5 trillion in 2022, 17% of the U.S. economy, placing an increasing burden on both the healthcare system and patients^1–4^. Strengthening primary care is a key strategy to mitigate these costs, as it reduces expensive downstream care through prevention, chronic disease management, and care coordination^5,6^. However, the effectiveness of primary care is threatened by a shortage of over 13,000 healthcare workers^7^, compounded by increasing demands from a growing, aging population with a rising burden of chronic diseases^8^. This imbalance limits access, increases wait times, and raises provider workload, ultimately driving up costs and straining the system^9–12^.

The growing imbalance between demand and capacity directly affects the day-to-day experience of primary care providers. Clinicians are increasingly tasked with managing larger, more complex patient panels while navigating substantial administrative burdens^13,14^. Providers now spend nearly twice as much time with electronic health records (EHRs) and completing desk work as they do with patients^15^. These demands contribute to high burnout rates, marked by emotional exhaustion and declining job satisfaction, which fuel early retirement and workforce attrition^15–18^. The cycle of rising demand and shrinking capacity jeopardizes the sustainability of primary care, underscoring the urgent need for solutions that reduce provider burden and support long-term system resilience.

Recent advances in artificial intelligence (AI) and machine learning (ML) are being integrated into primary care settings to alleviate workload and improve operational efficiency^19^. These technologies assist with diverse aspects of care, including risk stratification, scheduling optimization, and early identification of high-risk patients for targeted interventions^20–24^. The emergence of generative AI and large language models has further reduced documentation burdens, such as ambient scribes and automated in-basket replies to patients, thereby freeing up clinician time and improving patient satisfaction^25–27^.

Despite these promising developments, most ML applications in primary care remain narrowly focused on disease-specific risk prediction, such as dementia^28^, cancer^21^, or opioid use^29^, rather than addressing broader system-level challenges. Existing patient-level utilization prediction models have primarily focused on acute care settings, such as length of care^30^ or 30-day readmissions^31^, or have limited their scope to face-to-face encounters^32^. While valuable, these approaches may not capture the full spectrum of patient demand in primary care, where the adoption of mature EHR technology has shifted many interactions to asynchronous or non-face-to-face formats such as patient messaging.

To address this gap, we developed the Friedman Score, an ML model that predicts total primary care utilization at the individual patient level, encompassing both face-to-face and non-face-to-face demands. The model is named in honor of the late Dr. Lawrence Friedman of UCSD Health, recognizing his leadership and contributions to advancing value-based primary care and promoting high-quality, cost-effective healthcare delivery. Following his dedication to improve value-based care, this model was created with the vision to address real-life challenges in clinical practice related to the supply and demand of healthcare time. By identifying patients likely to require high levels of provider time in the upcoming year, the model supports data-guided proactive planning strategies, including informing strategies to balance physician panel sizes for more equitable workload distribution, providing additional follow-up for patients with complex needs, and scheduling earlier or more frequent visits to address emerging risks before they escalate to decrease downstream costs. This approach offers a pathway to enhance operational efficiency, reduce provider burden, and strengthen the long-term resilience of primary care.

## Results

### Data characteristics

The final feature set consisted of 39 variables, grouped into categories: age, diseases, procedures, medications, acute visits, and social determinants of health (SDoH). The 2022 dataset included 75,143 patients, and the 2023 dataset included 78,216 patients. Variable distributions for both cohorts are shown in Table 1. Estimated total primary care time utilization increased from 125,677 h (1.67 h per patient per year) in 2022 to 138,185 h (1.77 h per patient per year) in 2023. Among low-use patients, the average utilization was 73 min in 2022 and 77 min in 2023. High Use patients averaged 196 min in 2022 and 208 min in 2023. Very High Use patients averaged 363 min in 2022 and 391 min in 2023. Supplementary Tables 4 and 5 present variable distributions by utilization groups for the 2022 and 2023 datasets.

**Table 1:** Data characteristics of the 2022 and 2023 patient cohorts

Variables		2022 (*N* = 75,143)	2023 (*N* = 78,216)
Age, years, median (IQR)		55 (38, 71)	55 (38, 70)
Sex, no. (%)	Female	42,264 (56.24)	44,086 (56.36)
	Male	32,855 (43.72)	34,089 (43.58)
	Unknown	24 (0.03)	41 (0.05)
Race, no. (%)	American Indian or Alaska Native	374 (0.50)	350 (0.45)
	Asian	11,314 (15.06)	12,225 (15.63)
	Black or African American	2961 (3.94)	3038 (3.88)
	Native Hawaiian or Other Pacific Islander	407 (0.54)	416 (0.53)
	Other race or mixed race	11,680 (15.54)	12,122 (15.50)
	White	46,546 (61.94)	47,749 (61.05)
	Unknown (patient cannot or refuses to declare race)	1861 (2.48)	2316 (2.96)
Ethnicity, no. (%)	Non-Hispanic	61,826 (82.28)	64,192 (82.07)
	Hispanic	10,948 (14.60)	11,276 (14.42)
	Unknown or unreported	2349 (3.12)	2748 (3.51)
Healthy places index, percentile	Reported, median (IQR)	74.27 (55.02, 87.24)	76.31 (55.55, 87.59)
	Unreported, no. (%)	2427 (0.03)	2013 (0.03)
Utilization (min), median (IQR)	Face-to-face utilization	60 (40, 95)	65 (45, 105)
	Non-face-to-face utilization	16 (8, 31)	15 (7, 28)
	Total utilization	78 (49, 124)	81 (50, 132)
Disease and procedures, no. (%)	Cancer	8479 (11.28)	8664 (11.08)
	Chronic heart failure	2681 (3.57)	2961 (3.79)
	Chronic liver disease	2467 (3.28)	2991 (3.82)
	Cirrhosis	738 (0.98)	749 (0.96)
	End-stage liver disease	719 (0.96)	821 (1.05)
	Chronic obstructive pulmonary disease	2074 (2.76)	2185 (2.79)
	Chronic kidney disease	5046 (6.72)	5355 (6.85)
	Cardiovascular disease	7152 (9.52)	7827 (10.01)
	Depression	15,443 (20.55)	16,659 (21.3)
	Schizophrenia	36 (0.05)	36 (0.05)
	Bipolar disorder	180 (0.24)	176 (0.23)
	Diabetes	7625 (10.15)	8316 (10.63)
	Dialysis	166 (0.22)	170 (0.22)
	HIV	422 (0.56)	425 (0.54)
	Hypertension	23,661 (31.49)	25,231 (32.26)
	Major organ transplant	29 (0.04)	31 (0.04)
	Palliative care	614 (0.82)	663 (0.85)
	Asthma	6741 (8.97)	7160 (9.15)
	Inflammatory bowel disease	875 (1.16)	941 (1.20)
	Sleep apnea	8298 (11.04)	9556 (12.22)
	Memory disorder	922 (1.23)	1177 (1.50)
	Sickle cell	107 (0.14)	20 (0.03)
	Non-invasive ventilation	63 (0.08)	64 (0.08)
	Hemorrhage	1446 (1.92)	1612 (2.06)
	Osteoporosis	88 (0.12)	1054 (1.35)
	Heart failure with reduced ejection fraction	573 (0.76)	286 (0.37)
	Coronary artery bypass grafting	1564 (2.08)	1711 (2.19)
	Percutaneous coronary intervention	440 (0.59)	470 (0.60)
Medications	Number of outpatients' meds, median (IQR)	5 (1, 10)	5 (2, 10)
	Immunosuppressant, no. (%)	510 (0.68)	484 (0.62)
	Antiplatelet med, no. (%)	9638 (12.83)	9857 (12.60)
	Anticoagulation med, no. (%)	4823 (6.42)	5148 (6.58)
	Long-acting opioid, no. (%)	6151 (8.19)	6495 (8.30)
Acute Visits	Number of hospital admissions in the past 180 days (patient-level), median (IQR)	0 (0, 0)	0 (0, 0)
	Number of ER visits in the past year (patient-level), median (IQR)	0 (0, 0)	0 (0, 0)
	Had emergency general surgery in the past 180 days, no. (%)	88 (0.12)	100 (0.13)
Social determinants of health	Social determinant of health score^62^, median (IQR)	0 (0, 1)	1 (1, 2)
	Homeless in the past year, no. (%)	187 (0.25)	214 (0.27)

Categorical variables are summarized as frequency and percentage of cases, expressed as no (%). Continuous variables are summarized as median and interquartile range (25th–75th percentile) of the entire study population, expressed as median (IQR).

The training dataset included 52,600 patients, the calibration dataset 11,271 patients, and the 2022 test dataset 11,272 patients. After excluding patients who appeared in the 2022 dataset, the 2023 temporal test dataset consisted of 23,643 patients, 30.2% of the total 2023 cohort. The remaining 54,573 patients who appeared in both years comprised the 2023 overlap dataset.

### Model performance

**Fig. 1:**
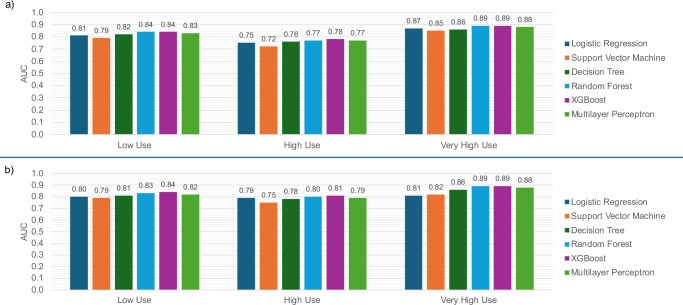
Area under the receiver operating characteristic curve (AUC) for each utilization class across six machine learning models (logistic regression, support vector machine, decision tree, random forest, eXtreme Gradient Boosting (XGBoost), and multilayer perceptron). **a** AUC for the 2022 testing dataset. **b** AUC for the 2023 temporal testing dataset.

Across both the 2022 and 2023 test sets, XGBoost and random forest demonstrated the strongest overall performance, achieving comparable and consistently high area under the receiver operating characteristic curve (AUC) values (Table 2). As shown in Fig. 1, all models demonstrated lower discriminative ability for the High Use group, reflecting the greater difficulty of classifying this middle category compared with the Low and Very High Use groups. Despite this challenge, tree-based ensemble methods, particularly XGBoost and random forest, maintained the strongest performance across all utilization classes in both test sets.

**Table 2:** Area under the receiver operating characteristics curve (AUC) with corresponding 95% confidence intervals before calibration for all datasets

		Logistic regression	Support vector machine	Decision tree	Random forest	XGBoost	Multilayer perceptron
2022	Training	0.804 (0.799, 0.809)	0.781 (0.776, 0.786)	0.827 (0.820, 0.828)	0.856 (0.853, 0.860)	0.840 (0.836, 0.844)	0.819 (0.815, 0.823)
	Testing	0.810 (0.800, 0.821)	0.789 (0.778, 0.799)	0.814 (0.803, 0.821)	0.833 (0.825, 0.842)	0.835 (0.826, 0.843)	0.824 (0.816, 0.832)
2023	Overlap Cohort	0.798 (0.785, 0.810)	0.785 (0.774, 0.796)	0.818 (0.807, 0.827)	0.842 (0.834, 0.850)	0.847 (0.839, 0.855)	0.833 (0.826, 0.840)
	Temporal Testing	0.807 (0.802, 0.810)	0.780 (0.775, 0.784)	0.797 (0.785, 0.793)	0.821 (0.817, 0.825)	0.822 (0.818, 0.826)	0.809 (0.805, 0.818)

Following isotonic regression, calibration improved across all models, with reductions in expected calibration error (ECE) while AUC remained stable (Table 3). For the 2022 test set, XGBoost achieved a similar AUC to the random forest model (*p* > 0.05) but outperformed all other algorithms. In the 2023 temporal test set, XGBoost demonstrated the highest AUC, with statistically significant improvements compared to all other models (*p* < 0.05). Detailed calibrated AUC results for all datasets are provided in Supplementary Table 6.

**Table 3:** Area under the receiver operating characteristics curve (AUC) and multiclass expected calibration error (ECE) on 2022 testing and 2023 temporal testing datasets

		Logistic regression	Support vector machine	Decision tree	Random forest	XGBoost	Multilayer perceptron
2022	Uncalibrated AUC	0.810	0.789	0.814	0.833	0.835	0.824
	Uncalibrated ECE	0.019	0.050	0.007	0.010	0.008	0.033
	Calibrated AUC	0.8062	0.788	0.814	0.833	0.833	0.823
	Calibrated ECE	0.014	0.049	0.006	0.009	0.005	0.009
2023	Uncalibrated AUC	0.798	0.785	0.818	0.842	0.847	0.833
	Uncalibrated ECE	0.030	0.090	0.031	0.030	0.026	0.019
	Calibrated AUC	0.791	0.783	0.818	0.841	0.846	0.832
	Calibrated ECE	0.034	0.061	0.031	0.023	0.024	0.026

Bolded values indicate the selected model’s (XGBoost) area under the receiver operating characteristic curve (AUC). Bold formatting was removed to improve visualization.

Supplementary Fig. 1 shows the F1 scores for XGBoost across a range of classification thresholds. Optimal thresholds were 0.46 for Low Use, 0.17 for High Use, and 0.20 for very High Use. To prevent multiple class assignments for a single patient, labeling safeguards were implemented. When adjacent classes exceeded their thresholds (e.g., both Low and High Use), the lower utilization class was assigned. In cases of conflicting classifications (e.g., both Low and Very High Use) or when all three classes were positive, the patient was labeled as “NA,” indicating insufficient certainty for assignment. This affected 1.8% of patients in the 2022 test set and 1.2% in the 2023 temporal test set. Using this labeling strategy, we evaluated a binary classification of Low Use vs High and Very High Use, aligning with the clinical goal of identifying high-utilization patients. For the 2022 test set, the model achieved an accuracy of 86.9%, sensitivity of 22.1%, specificity of 97.9%, and a PPV of 64.7%. On the 2023 temporal test set, accuracy was 92.8%, sensitivity 15.5%, specificity 98.5%, and PPV 43.2%. Full performance metrics for all models are provided in Supplementary Table 7.

### Model interpretability analysis

**Fig. 2:**
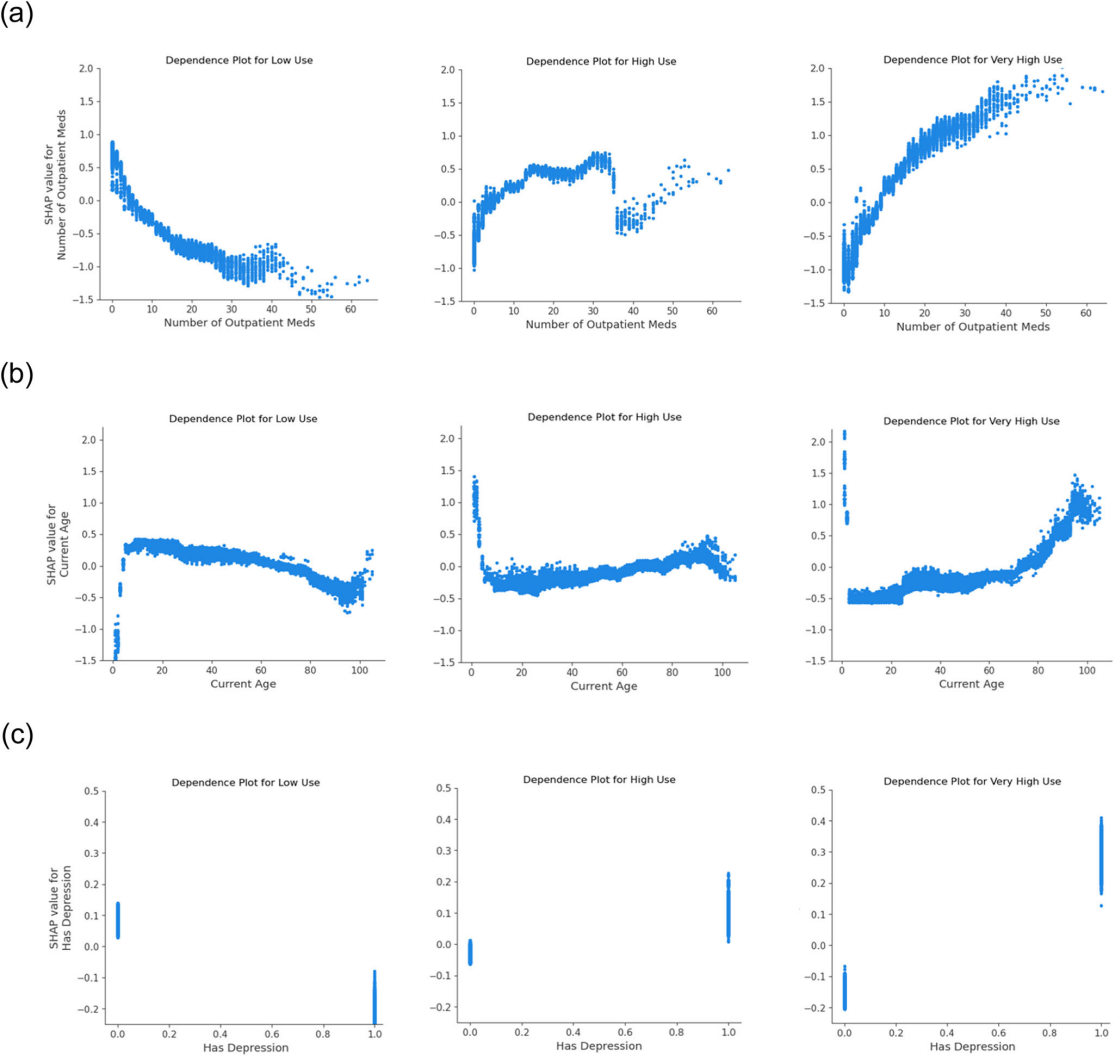
SHAP dependence plots showing the relationship between feature and model classification for each utilization class: Low Use (left), High Use (middle), and Very High Use (right). The y-axis represents SHAP values, where higher values indicate a greater likelihood of classification into the given utilization class, and lower values indicate a lower likelihood. **a** Number of outpatient medications. **b** Age. **c** Depression.

SHAP analysis of the XGBoost model, shown in Supplementary Fig. 2, identified the top five features with the highest mean SHAP values across the three utilization classes: number of outpatient medications, age, depression, number of emergency room (ER) visits in the past year, and hypertension. Among these, the number of outpatient medications was the most predictive feature across all classes. Figure 2 provides detailed SHAP value trends for the number of outpatient medications, age, and depression, stratified by utilization class. As the number of outpatient medications and patient age increase, the likelihood of higher utilization also increases. Similarly, patients with depression were more likely to have higher overall utilization.

## Discussion

In this study, we developed and validated the Friedman Score, an ML model to predict primary care utilization at the patient level. We introduced a novel outcome metric, total primary care utilization, that accounts for both face-to-face and non-face-to-face clinical effort. Using data from over 98,000 patients, XGBoost demonstrated high discriminative ability and robust calibration for identifying high-utilization patients compared with benchmark algorithms. SHAP analysis confirmed that clinically meaningful predictors, including medication burden, age, and depression, drove model performance, reflecting both established and less traditionally recognized contributors to primary care demand.

Between 2022 and 2023, primary care utilization increased by 10%, with consistent growth across all patient groups. This rise likely reflects the effects of a growing and aging population, an increasing burden of chronic disease, particularly in the post-COVID period, and, more importantly, a rebound in care-seeking as the impact of the SARS-CoV-2 pandemic subsided^33,34^. Differences across utilization groups highlight diverse patient needs: High Use patients required 2.7 times more total primary care team time than Low Use patients, and Very High Use patients required 5 times more. Higher-utilization groups were characterized by older age, greater chronic disease burden (e.g., depression, chronic heart failure, diabetes), and higher medication use. Very High Use patients frequently had complex needs such as hospital stays and long-acting opioid prescriptions, underscoring their demand for intensive and continuous care.

Utilization patterns also reflected known equity-related differences. To better understand demographic and social determinants associated with utilization risk, utilization time was analyzed across utilization categories. Patients with higher social vulnerability demonstrated greater predicted time utilization. Women were more likely to consume higher levels of care, consistent with prior literature^35,36^. Black or African American patients were overrepresented in the Very High Use group, while Asian patients were underrepresented. Very High Use patients also had lower Healthy Places Index (HPI) percentiles^37^, indicating greater contextual barriers such as housing, transportation, and education, which aligns with established SDoH^38^. These findings are important because the interventions informed by this model are designed to improve the efficiency of care delivery while recognizing that patients with the highest predicted risk are often those experiencing greater health disparities. Ongoing evaluation is needed to prospectively assess the impact of these interventions to ensure that existing gaps are not widened. Our implementation strategy specifically aims to proactively engage patients classified as High or Very High Use by the Friedman Score through multidisciplinary care and targeted attention to SDoH.

All models showed reduced performance when classifying the High Use group, reflecting the overlap between adjacent categories. Nevertheless, XGBoost outperformed or matched all benchmarks across both years, demonstrating robustness in handling these nuanced distinctions. SHAP analysis reinforced the clinical plausibility of the model: medication burden strongly predicted high utilization^39,40^, young pediatric and elderly patients were more likely to fall into higher utilization categories^41–44^, and depression emerged as a stronger predictor of Very High Use than common chronic conditions like hypertension. This may reflect the central role of primary care in managing mental health needs^45,46^ and suggest that supporting mental health care could relieve some utilization pressure on primary care.

The Friedman Score’s value lies not only in its predictive power but in its integration into clinical workflows to proactively manage complex patient needs. After validation and a health equity review, the score was embedded into the Epic EHR alongside the ratio of non–face-to-face to face-to-face utilization from the prior year to provide easy visibility for clinicians and to support real-time decision-making. In early implementation, care team members, such as nurse practitioners (NPs), review the score in daily schedules, identify high-risk patients, and coordinate additional support. Prior literature has demonstrated that pharmacists can be involved in visits to help manage complex care needs^47^, and future adaptations of the Friedman Score may extend to coordinating additional services from pharmacy and mental health. The utilization ratio further helps identify patterns of care among patients with high non-face-to-face activity (e.g., frequent patient portal messages or phone calls), helping clinicians better understand the needs of the patient when the patient is between billable encounters. Our current pilot expands from reactive support to proactive outreach: a dedicated NP and MA dyad conduct structured outreach phone calls and messages to Very High Use patients following their clinic visits to assess unmet needs (i.e., health literacy, medication access, mental health, etc.), confirm understanding of treatment plans, and coordinate appropriate follow-up with the primary care team. This redesign enables anticipatory care, reduces provider burden through targeted resource allocation, and may improve overall system efficiency.

Beyond these immediate workflow enhancements, the Friedman Score provides a powerful tool for health system leadership. At a macro level, it enables a shift from reactive care to a data-driven, population health management strategy. By aggregating risk data, health systems can forecast demand, inform staffing strategies, and justify the allocation of specialized resources such as NPs, social workers, or pharmacists. This predictive capability may be crucial for success in value-based care arrangements, where managing the costs and outcomes of high-utilization populations is paramount. Ultimately, the score can serve as a foundational element for aligning clinical operations with financial sustainability, allowing the health system to optimize capacity, improve care quality, and reduce the total cost of care for its most complex patient populations.

This study has limitations. Because the data originated from a single academic health system and relied on internal registry metrics specific to this academic medical center, the model may not generalize to institutions with different EHR configurations, patient populations, or coding practices. These registries reflect quality program-derived population cohorts that are likely comparable to those used in other health systems engaged in public reporting. Patients with a longer longitudinal care within the system may also have more complete data, potentially leading to differences in data richness and model performance across patient subgroups. Nevertheless, integrated and interoperable data environments are becoming increasingly common, which may improve the generalizability of such approaches^48–50^. In addition, time estimates for some face-to-face and non–face-to-face activities had to be derived using local expert input rather than standardized benchmarks, which may introduce error. There remains a gap in the literature regarding standardized, contemporary estimates of task-level time measurement^51–54^. As workflows evolve and more precise measures become available, time estimates may shift, potentially affecting the model’s long-term accuracy and applicability.

In conclusion, the Friedman Score provides a robust, interpretable framework for predicting primary care utilization, with XGBoost outperforming or matching benchmark algorithms across evaluation periods. For patients, the model enables more personalized, proactive care; for care teams, it can enhance patient management and reduce provider burden; and for health systems, it may provide a foundation for strategic planning and resource allocation. As healthcare systems face mounting demands, predictive tools such as the Friedman Score can be critical for ensuring sustainable, high-quality, and efficient care delivery.

## Methods

### Method overview

We conducted a retrospective cohort study using structured EHR data from primary care patients at UCSD Health for calendar years 2022 and 2023 to develop a predictive tool for primary care utilization. We defined a novel outcome metric, total primary care utilization, which estimates both face-to-face and non-face-to-face clinical time. Predictor variables included age, chronic conditions, procedures, medication history, acute care encounters, and social determinants of health. We selected eXtreme Gradient Boosting (XGBoost) as the primary algorithm and benchmarked its performance against five commonly used supervised ML methods. This project was reviewed and approved by the UCSD ACQUIRE (Aligning and Coordinating Quality Improvement, Research, and Evaluation) Committee, which is delegated by the UCSD Institutional Review Board (IRB) to determine when projects do not require IRB oversight. The ACQUIRE Committee determined that this project was intended for local improvement of care and clinical practice at UCSD Health and does not meet the regulatory definition of human subjects research under 45 CFR 46 or 21 CFR 56. Therefore, IRB review and approval were not required.

The study’s risk of bias was evaluated using the PROBAST + AI (Prediction model Risk Of Bias Assessment Tool for Artificial Intelligence) guidelines (Supplementary Table 8)^55^. This manuscript was prepared in accordance with the TRIPOD + AI (Transparent Reporting of a multivariable prediction model for Individual prognosis or diagnosis—artificial intelligence) reporting guideline (Supplementary Table 9)^56^.

### Study setting and participants

This study included active patients from UCSD Health’s primary care departments, including family medicine, internal medicine, geriatrics, and pediatrics, between 2022 and 2023. Active patients were defined as those with at least one billable encounter in the primary care department during the study year, documented using one of 77 primary care-specific current procedural terminology (CPT) codes (Supplementary Table 1). These CPT codes align with organizational logic reviewed by auditors for public reporting and are consistent with the value sets used in quality reporting programs. Obstetrics, gynecology, and reproductive health services were excluded, consistent with jurisdictional Centers for Medicare & Medicaid Services (CMS) public reporting guidance for primary care quality programs. Patients who expired during the study period were included if they had received care, and no exclusion criteria were applied. Two annual datasets were constructed: one for patients active from January to December 2022, and another for those active during the same timeframe in 2023.

### Model outcomes

The model classified patients into three primary care utilization classes based on yearly total utilization time in minutes: Low Use (0–85th percentile), High Use (>85–95th percentile), and Very High Use (>95th percentile). These thresholds were informed by Medicare expenditure patterns under the value-based care framework, where approximately 5% of patients account for nearly 50% of total healthcare expenditures^57,58^. Because this group often has limited opportunity for modifiable intervention that affects expenditures, Medicare value-based payment programs frequently focus on the “rising-risk” tier, patients in the next highest expenditure group, where proactive care management can have a greater impact on cost^59,60^. Therefore, this model was designed to identify two tiers of time utilization to support differentiated care strategies and targeted resource allocation within primary care. Total utilization was calculated by summing face-to-face and non-face-to-face time by the clinical teams in primary care. Work outside primary care in terms of encounters was not included in the time attribution.

Face-to-face utilization included in-person and telemedicine encounters, identified by outpatient CPT codes from UCSD Health primary care departments. Each CPT code was mapped to a time duration as defined in the CPT definition; when no explicit time was available, a time value estimate was consistently applied for the work type guided by real-world task estimates (Supplementary Table 1). In general, brief services were assigned 15 min, moderate services 30 min, and complex services 60 min, following general web-based clinical service guidance^61^. For example, CPT codes 99381–99387 (new patient preventive medicine services) were mapped to 30 min. A patient’s face-to-face utilization was computed as the sum of encounter volumes weighted by encounter duration.

Non-face-to-face utilization captured indirect clinical work, such as telephone calls, order placements (e.g., labs, imaging, referrals), prescription refills, and direct patient messages. As existing literature lacks time estimates for individual non-face-to-face tasks, we estimated weights based on the relative time burden of each task that represented the collective primary care team time, including medical assistants (MAs), registered nurses (RN), pharmacists, and clinicians, etc. A telephone call or patient portal message was assigned a weight of 2 min, while a prescription refill or order placement was assigned a weight of 1 min. Patient-level non-face-to-face utilization was then calculated by summing task volumes weighted by these estimates.

### Predictor variable selection

Candidate predictor variables were initially drawn from an existing primary care risk score in use at UCSD Health, which included age, chronic conditions, and acute visits. This custom risk score was developed in 2015 and is still in use today to predict the top 5% of Medicare spend. To broaden the feature set for this use case, we incorporated variables from established hospitalization and pharmacy risk models (e.g., Charlson Comorbidity Index, LACE+), capturing additional comorbidities and medications. We further expanded the list using pre-built, EHR-based real-time registries or registry metrics for chronic conditions and social determinants of health (SDoH). The local EHR has more than 180 active registries that include diabetes, hypertension, HIV, etc. The Epic SDoH score incorporates patient-reported social and environmental factors that influence health status, such as housing stability, food security, and transportation access^62^. The final set of predictors was determined by data completeness, availability across both study years, and recency, as verified through Epic SlicerDicer. Predictors were organized into categories: age, diseases, procedures, medications, acute visits, and SDoH. A detailed list of predictive variables is provided in Supplementary Table 2.

To reduce potential bias, variables directly reflecting diversity, equity, and inclusion (DEI) domains, including gender, race, sexual orientation, ethnicity, and Healthy Places Index (HPI)^37^, were intentionally excluded from model training. Unlike the patient-reported SDoH score, the HPI is a composite measure of community health and well-being that integrates socioeconomic, environmental, educational, and housing indicators to quantify neighborhood-level opportunity for better health^37^.

### Data preprocessing and profiling

**Fig. 3:**
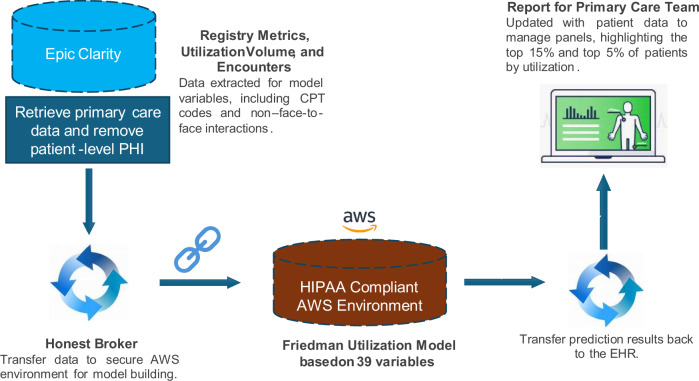
Overview of the data flow and model pipeline for the Friedman Model. Primary care patient data, including registry metrics, CPT codes, and non-face-to-face interactions, are extracted from the Epic Clarity database using SQL Server Management Studio (SSMS). Patient-level personal health information (PHI) is stripped before transfer to a secure HIPAA-compliant AWS environment via an Honest Broker. The model, developed in Python using 39 predictor variables, generates utilization predictions. Results are provided to the primary care team in regularly updated reports highlighting the top 15% and top 5% of high-utilization patients to support proactive panel management and targeted care interventions.

Two datasets, each containing identical sets of variables, including registry data, CPT codes, and non-face-to-face interaction, were extracted from the Epic Clarity database using SQL. To ensure patient privacy during model training, all datasets were stripped of direct personal health information (PHI). The datasets were transferred to a HIPAA-compliant Amazon Web Services (AWS) Virtual Research Desktop for preprocessing and model development. A detailed overview of the data flow and pipeline is shown in Fig. 3.

Patients in both datasets were categorized into utilization classes based on their total yearly primary care utilization. Exploratory data analysis was conducted across study years and within utilization groups for a given year. Continuous variables were summarized using medians and interquartile ranges (IQR), while categorical variables were reported as frequencies and percentages. For model training, all predictor variables were scaled between 0 and 1. Missing data were assigned default values of 0 or “No,” as appropriate. Continuous features were normalized to a 0-1 scale based on clinically meaningful maximum values determined through expert input (A.M.S.). For example, the SDoH score was capped at a maximum value of 20.

### Model development

The 2022 dataset was randomly split into training, calibration, and testing subsets using a 70:15:15 ratio, while preserving the original distribution of utilization classes. To evaluate the model’s temporal generalizability, we constructed a secondary test set from the 2023 dataset, consisting exclusively of patients who did not present in the 2022 cohort. Patients who appeared in both years were grouped into a separate overlap dataset for additional analysis.

We selected XGBoost as the primary model for predicting utilization classes due to its strong performance on structured tabular data and its ability to provide interpretable feature-level explanations^63,64^. For benchmarking, we trained and tuned five additional algorithms: logistic regression, support vector machine (SVM), decision tree, random forest, and multilayer perceptron (MLP), using exhaustive grid search and 10-fold cross-validation. To accommodate the three-class outcome, model configurations were tailored to each algorithm’s multiclass capabilities. Tree-based methods (decision tree, random forest, and XGBoost) and the MLP inherently support multiclass classification, whereas binary classifiers such as the SVM were extended using a one-vs-rest (OvR) approach. Logistic regression was implemented under a multinomial framework to directly model all classes simultaneously. Hyperparameters (Supplementary Table 3)^65–68^ were optimized for area under the receiver operating characteristic curve (AUC), and calibration was performed with isotonic regression^69,70^. Calibration quality was assessed using 15-fold multiclass expected calibration error (ECE)^71,72^. Model performance was evaluated using AUC on both the 2022 test set and 2023 temporal test set, with 95% confidence intervals (CIs) obtained through stratified bootstrapping. Model interpretability for XGBoost was further explored using SHAP (SHapley Additive exPlanations) analysis.

F1 scores were computed across different classification thresholds. The final classification threshold for each class was selected based on the point that maximized its respective F1 score^73^. Based on these thresholds, additional performance metrics, including accuracy, sensitivity, specificity, and positive predictive value (PPV), were calculated using a binary classification of Low Use vs High and Very High Use. This approach reflects the clinical priority of identifying high-utilization patients to better support physician decision-making.

All statistical analyses and model development were conducted using Python 3.11. Data cleaning, model training, calibration, and performance evaluation were performed using the following packages: *pandas* 1.5.3, *scikit-learn* 1.2.2, and *xgboost* 2.0.1. ECE was computed using *TorchMetrics* 1.1.2, and model interpretability analysis was conducted with *SHAP* 0.42.1.

## Supplementary information

Supplementary information

## Data Availability

Access to the de-identified UCSD cohort can be made available by contacting the corresponding author and via approval from the UCSD Institutional Review Boards (IRB) and Health Data Oversight Committee (HDOC).

## Code availability

The code used for model development is available from the corresponding author upon reasonable request.
