# Supplementary material for: Application of a machine learning model to predict the estimated primary care patient time consumption
Source: Npj Health Syst. 2026 Feb 2;3:12. doi: 10.1038/s44401-025-00061-0 (PMC12864026; doi:10.1038/s44401-025-00061-0)
Supplement: Supplementary file 1 — Supplementary information [file 44401_2025_61_MOESM1_ESM.pdf]

SUPPLEMENTARY INFORMATION

Supplementary Table 1. Primary care current procedural terminology (CPT) codes used in the study and estimated time duration. Times estimated using heuristic: 15 min = brief, 30 min = moderate, 60 min = extended.

CPT Codes	Estimated Time (min)
G0402	30
G0438	45
G0439	30
92002	10
92004	44
92012	24
92014	39
99201	10
99202	20
99203	30
99204	45
99205	60
99211	5
99212	10
99213	15
99214	25
99215	40
99304	25
99305	35
99306	45
99307	10
99308	15
99309	25
99310	35
99315	29
99316	31
99318	30
99324	20
99325	30
99326	45
99327	60
99328	75
99334	15

99335	25
99336	40
99337	60
99341	15
99342	30
99343	30
99344	60
99345	75
99346	30
99347	20
99348	30
99349	40
99350	60
99381	30
99382	30
99383	30
99384	30
99385	30
99386	30
99387	30
99391	45
99392	45
99393	45
99394	45
99395	45
99396	45
99397	45
99401	15
99402	30
99403	45
99404	60
99411	30
99412	60
99420	15
99429	15
99455	60
99456	60
99461	30
99490	20

99495	30
99496	60
99441	10
99442	20
99443	30

Supplementary Table 2. Final predictor variable selected for the model.

Category	Features
Age	Age
Diseases and Procedures	Cancer, Chronic heart failure, Chronic liver disease, Cirrhosis, End stage liver disease, Chronic obstructive pulmonary disease, Chronic kidney disease, Cardiovascular disease, Depression, Schizophrenia, Bipolar Disorder, Diabetes, Dialysis, HIV, Hypertension, Major organ transplant, Palliative care, Asthma, Inflammatory bowel disease, Sleep apnea, Memory disorder, Sickle cell, Non-invasive ventilation, Hemorrhage, Osteoporosis, Heart failure with reduced ejection fraction, Coronary artery bypass grafting, Percutaneous coronary intervention
Medications	Number of active outpatient meds, Immunosuppressant (active), Antiplatelet med (active), Anticoagulation med (active), Long acting opioid (active)
Acute Visits	Number of hospital admissions in the past 180 days, Number of ER visits in the past year, Had emergency general surgery in the past 180 days
Social Determinants of Health	Social determinant of health score, Homeless in past year

Supplementary Table 3. Hyperparameter ranges for model tuning.

Model	Hyperparameters	Ranges
Logistic regression	C	10^{-30}, 10^{-29}, …, 10^{2}
	penalty	none, l1, l2, elastic net
	l1_ratio for elastic net	0.1, 0.2, …, 0.9
Support vector machine	C	10^{-8}, 10^{-7}, …, 10^{1}
	kernel	linear
Decision tree	max_features	sqrt, log2, 0.3
	max_depth	10, 11, …, 30
	min_samples_split	10, 20, …, 100
	min_samples_leaf	10, 20, …, 60
Random forest	n_estimators	100, 200, 500, 1000
	max_features	sqrt, log2, 0.3
	max_depth	9, 12, …, 30
	min_samples_split	10, 20, …, 50
	min_samples_leaf	10, 15, …, 40
XGBoost	n_estimators	100, 200, 500, 1000, 2000
	max_depth	3, 4, …, 12
	learning_rate	0.001, 0.01, 0.1, 0.2, 0.3
	subsample	0.5, 0.6, …, 1.0
Multilayer perceptron neural network	hidden_layer	(20,), (30,), (30, 15), (40,), (40, 20), (50,), (50, 25), (60,), (60, 30)
	alpha	10^{-5}, 10^{-4}, …, 10^{-1}
	learning_rate	10^{-4}, 10^{-3}, 10^{-2}, 10^{-1}
	solver	adam, sgd

Supplementary Table 4. Patient characteristics for different utilization class in 2022. Categorical variables are summarized as frequency and percentage, expressed as no (%). Continuous variables are summarized as median and interquartile range (25th-75th percentile) of the entire study population, expressed as median (IQR).

**Variables**		**Utilization Class**		
		**Low (N = 63,712)**	**High (N = 7,713)**	**Very High (N=3,718)**
Age, years, median (IQR)		52 (36, 69)	68 (52, 77)	72 (59, 81)
Sex, no. (%)	Female	35609 (55.89)	4468 (57.93)	2187 (58.82)
	Male	28081 (44.07)	3243 (42.05)	1531(41.18)
	Unknown	22 (0.03)	2 (0.03)	0 (0)
Race, no. (%)	American Indian or Alaska Native	293 (0.46)	52 (0.67)	29 (0.78)
	Asian	10045 (15.77)	955 (12.38)	314 (8.45)
	Black or African American	2344 (3.68)	373 (4.84)	244 (6.56)
	Native Hawaiian or Other Pacific Islander	348 (0.55)	39 (0.51)	20 (0.54)
	Other Race or Mixed Race	9915 (15.56)	1200 (15.56)	565 (15.20)
	White	39043 (61.28)	4990 (64.70)	2513 (67.59)
	Unknown (Patient cannot or refuses to declare race)	1724 (2.71)	104 (1.35)	33 (0.89)
Ethnicity, no. (%)	Non-Hispanic	52236 (82.00)	6444 (83.55)	3136 (84.35)
	Hispanic	9316 (14.62)	1124 (14.57)	528 (14.20)
	Unknown or unreported	2150 (3.38)	145 (1.88)	54 (1.45)
Healthy Places Index, percentile	Reported, median (IQR)	75.35 (55.27, 87.39)	72.39 (51.76, 86.1)	69.49 (45.39, 85.56)
	Unreported, no. (%)	1755 (0.03)	338 (0.04)	334 (0.09)
Estimated Utilization in minutes, median (IQR)	Face-to-face	50 (30, 75)	150 (130, 170)	255 (215, 320)
	Non-face-to-face	13 (6, 24)	43 (29, 61)	70 (48, 102)
	Total	67 (46, 99)	192 (173, 216)	322.5 (281, 402.75)
Disease and Procedures, no. (%)	Cancer	6444 (10.11)	1287 (16.69)	748 (20.12)
	Chronic heart failure	1513 (2.37)	588 (7.62)	580 (15.60)
	Chronic liver disease	1685 (2.64)	451 (5.85)	331 (8.90)

	Cirrhosis	488 (0.77)	137 (1.78)	113 (3.04)
	End stage liver disease	482 (0.76)	138 (1.79)	99 (2.66)
	Chronic obstructive pulmonary disease	1179 (1.85)	468 (6.07)	427 (11.48)
	Chronic kidney disease	3161 (4.96)	1035 (13.42)	850 (22.86)
	Cardiovascular disease	4670 (7.33)	1458 (18.90)	1024 (27.54)
	Depression	4670 (7.33)	2523 (32.71)	1802 (48.47)
	Schizophrenia	26 (0.04)	5 (0.06)	5 (0.13)
	Bipolar Disorder	139 (0.22)	25 (0.32)	16 (0.43)
	Diabetes	5104 (8.01)	1606 (20.82)	915 (24.61)
	Dialysis	100 (0.16)	36 (0.47)	30 (0.81)
	HIV	325 (0.51)	62 (0.80)	35 (0.94)
	Hypertension	17198 (26.99)	3569 (46.27)	1399 (37.63)
	Major organ transplant	23 (0.04)	4 (0.05)	2 (0.05)
	Palliative care	354 (0.56)	95 (1.23)	165 (4.44)
	Asthma	5091 (7.99)	991 (12.85)	659 (17.72)
	Inflammatory bowel disease	683 (1.07)	125 (1.62)	67 (1.80)
	Sleep apnea	5678 (8.91)	1564 (20.28)	1056 (28.40)
	Memory disorder	515 (0.81)	205 (2.66)	202 (5.43)
	Sickle cell	86 (0.13)	14 (0.18)	7 (0.19)
	Non-invasive ventilation	40 (0.06)	12 (0.16)	11 (0.30)
	Hemorrhage	1048 (1.62)	235 (3.05)	163 (4.38)
	Osteoporosis	48 (0.08)	22 (0.29)	18 (0.48)
	Heart failure with reduced ejection fraction	367 (0.58)	110 (1.43)	96 (2.58)
	Coronary artery bypass grafting	1014 (1.59)	316 (4.10)	234 (6.29)
	Percutaneous coronary intervention	270 (0.42)	84 (1.09)	86 (2.31)
Medications	Number of outpatient meds, median (IQR)	4 (1, 8)	11 (6, 16)	15 (10, 21)
	Immunosuppressant, no. (%)	339 (0.53)	101 (1.31)	70 (1.88)
	Antiplatelet med, no. (%)	6978 (10.95)	1663 (21.56)	997 (26.82)
	Anticoagulation med, no. (%)	3079 (4.83)	968 (12.55)	776 (20.87)
	Long acting opioid, no. (%)	3711 (5.82)	1324 (17.17)	1116 (30.02)

Acute Visits	Number of hospital admissions in the past six months, median (IQR)	0 (0, 0)	0 (0, 0)	0 (0, 0)
	Number of ER visits in the past six months, median (IQR)	0 (0, 0)	0 (0, 0)	0 (0, 1)
	Had emergency general surgery, no. (%)	60 (0.09)	12 (0.16)	16 (0.43)
Social Determinants of Health	Social determinant of health score, median (IQR)	0 (0, 1)	0 (0, 1)	0 (0, 2)
	Homeless in past year, no. (%)	127 (0.20)	34 (0.44)	26 (0.70)

Supplementary Table 5. Patient characteristics for different utilization class in 2023. Categorical variables are summarized as frequency and percentage, expressed as no (%). Continuous variables are summarized as median and interquartile range (25th-75th percentile) of the entire study population, expressed as median (IQR).

Variables		Utilization Class		
		Low (N = 66,445)	High (N = 7,876)	Very High (N=3,895)
Age, years, median (IQR)		52 (36, 68)	68 (53, 77)	72 (60, 81)
Sex, no. (%)	Female	37038 (55.74)	4725 (59.99)	2323 (59.64)
	Male	29369 (44.20)	3149 (39.98)	1571 (40.33)
	Unknown	38 (0.06)	2 (0.03)	1 (0.03)
Race, no. (%)	American Indian or Alaska Native	278 (0.42)	48 (0.61)	24 (0.62)
	Asian	10874 (16.37)	961 (12.20)	390 (10.01)
	Black or African American	2466 (3.71)	339 (4.30)	233 (5.98)
	Native Hawaiian or Other Pacific Islander	350 (0.53)	42 (0.53)	24 (0.62)
	Other Race or Mixed Race	10276 (15.47)	1247 (15.83)	599 (15.38)
	White	40036 (60.25)	5122 (65.03)	2591 (66.52)
	Unknown (Patient cannot or refuses to declare race)	2165 (3.26)	117 (1.49)	34 (0.87)
Ethnicity, no. (%)	Non-Hispanic	54385 (81.85)	6522 (82.81)	3285 (84.34)
	Hispanic	9555 (14.38)	1171 (14.87)	550 (14.12)
	Unknown or unreported	2505 (3.77)	183 (2.32)	60 (1.54)
Healthy Places Index, percentile	Reported, median (IQR)	76.61 (55.74, 87.98)	72.39 (51.76, 86.1)	71.27 (48.11, 85.67)
	Unreported, no. (%)	1659 (0.02)	338 (0.04)	159 (0.04)
Estimated Utilization in minutes, median (IQR)	Face-to-face	55 (40, 85)	160 (145, 185)	285 (240, 355)
	Non-face-to-face	12 (6, 22)	40 (26, 56)	62 (42, 89)
	Total	71 (47, 104)	203 (184, 229)	345 (299, 429)
Disease and Procedures, no. (%)	Cancer	6560 (9.87)	1371 (17.41)	733 (18.82)
	Chronic heart failure	1632 (2.46)	661 (8.39)	668 (17.15)
	Chronic liver disease	2110 (3.18)	532 (6.75)	349 (8.96)

	Cirrhosis	492 (0.74)	142 (1.80)	115 (2.95)
	End stage liver disease	547 (0.82)	154 (1.96)	120 (3.08)
	Chronic obstructive pulmonary disease	1198 (1.80)	512 (6.50)	475 (12.20)
	Chronic kidney disease	3333 (5.02)	1113 (14.13)	909 (23.34)
	Cardiovascular disease	5099 (7.67)	1563 (19.85)	1166 (29.94)
	Depression	11978 (18.03)	2794 (35.47)	1887 (48.45)
	Schizophrenia	31 (0.05)	5 (0.06)	0 (0.00)
	Bipolar Disorder	136 (0.20)	23 (0.29)	17 (0.44)
	Diabetes	5570 (8.38)	1714 (21.76)	1032 (26.50)
	Dialysis	107 (0.16)	29 (0.37)	34 (0.87)
	HIV	338 (0.51)	53 (0.67)	34 (0.87)
	Hypertension	18296 (27.54)	3518 (44.67)	1318 (33.84)
	Major organ transplant	28 (0.04)	2 (0.03)	1 (0.03)
	Palliative care	371 (0.56)	137 (1.74)	155 (3.98)
	Asthma	5429 (8.17)	1068 (13.56)	663 (17.02)
	Inflammatory bowel disease	742 (1.12)	125 (1.59)	74 (1.90)
	Sleep apnea	6563 (9.88)	1796 (22.80)	1197 (30.73)
	Memory disorder	662 (1.00)	274 (3.48)	241 (6.19)
	Sickle cell	17 (0.03)	0 (0.00)	3 (0.08)
	Non-invasive ventilation	39 (0.06)	10 (0.13)	15 (0.39)
	Hemorrhage	1186 (1.78)	231 (2.93)	195 (5.01)
	Osteoporosis	651 (0.98)	233 (2.96)	170 (4.36)
	Heart failure with reduced ejection fraction	185 (0.28)	60 (0.76)	41 (1.05)
	Coronary artery bypass grafting	1125 (1.69)	324 (4.11)	262 (6.73)
	Percutaneous coronary intervention	279 (0.42)	89 (1.13)	102 (2.62)
Medications	Number of outpatient meds, median (IQR)	4 (1, 9)	11 (7, 16)	16 (10, 22)
	Immunosuppressant, no. (%)	328 (0.49)	97 (1.23)	59 (1.51)
	Antiplatelet med, no. (%)	7105 (10.69)	1663 (21.11)	1089 (27.96)
	Anticoagulation med, no. (%)	3262 (4.91)	1026 (13.03)	860 (22.08)

	Long acting opioid, no. (%)	3907 (5.88)	1435 (18.22)	1153 (29.60)
Acute Visits	Number of hospital admissions in the past six months, median (IQR)	0 (0, 0)	0 (0, 0)	0 (0, 0)
	Number of ER visits in the past six months, median (IQR)	0 (0, 0)	0 (0, 0)	0 (0, 1)
	Had emergency general surgery, no. (%)	59 (0.09)	17 (0.22)	24 (0.62)
Social Determinants of Health	Social determinant of health score, median (IQR)	1 (1, 2)	1 (0, 2)	2 (0, 3)
	Homeless in past year, no. (%)	151 (0.23)	34 (0.43)	29 (0.74)

Supplementary Table 6. Area under the receiver operating characteristics curve (AUC) with corresponding 95% confidence intervals after calibration for all datasets.

		**Logistic Regression**	**Support Vector Machine**	**Decision Tree**	**Random Forest**	**XGBoost**	**Multilayer Perceptron**
**2022**	**Training**	0.801 (0.796, 0.805)	0.780 (0.775, 0.784)	0.823 (0.818, 0.827)	0.856 (0.852, 0.860)	0.839 (0.835, 0.843)	0.824 (0.820, 0.828)
	**Testing**	0.806 (0.796, 0.817)	0.788 (0.778, 0.798)	0.811 (0.803, 0.813)	0.833 (0.824, 0.841)	0.833 (0.825, 0.841)	0.823 (0.815, 0.832)
**2023**	**Overlap Cohort**	0.796 (0.792, 0.800)	0.778 (0.773, 0.782)	0.790 (0.786, 0.794)	0.824 (0.817, 0.824)	0.821 (0.818, 0.825)	0.812 (0.808, 0.815)
	**Temporal Testing**	0.791 (0.779, 0.803)	0.783 (0.772, 0.793)	0.820 (0.810, 0.829)	0.841 (0.832, 0.849)	0.846 (0.838, 0.854)	0.832 (0.823, 0.840)

Supplementary Table 7a. Binary classification performance (Low Use vs. High and Very High Use) for the 2022 testing set.

	Logistic Regression	Support Vector Machine	Decision Tree	Random Forest	XGBoost	Multilayer Perceptron
**NA (%)**	2.07	6.8	2.25	2.33	1.80	0.00
**Accuracy**	0.870	0.880	0.865	0.871	0.869	0.860
**Balanced Accuracy**	0.607	0.545	0.562	0.605	0.600	0.629
**Sensitivity**	0.236	0.095	0.137	0.231	0.221	0.297
**Specificity**	0.978	0.995	0.987	0.978	0.979	0.961
**PPV**	0.648	0.749	0.637	0.640	0.647	0.579
**NPV**	0.883	0.882	0.873	0.884	0.881	0.884
**F1**	0.346	0.169	0.226	0.340	0.330	0.393

Supplementary Table 7b. Binary classification performance (Low Use vs. High and Very High Use) for the 2023 temporal testing set.

	Logistic Regression	Support Vector Machine	Decision Tree	Random Forest	XGBoost	Multilayer Perceptron
**NA (%)**	1.04	3.01	1.11	1.38	1.15	0.00
**Accuracy**	0.929	0.935	0.931	0.929	0.928	0.921
**Balanced Accuracy**	0.552	0.515	0.525	0.561	0.570	0.595
**Sensitivity**	0.116	0.034	0.055	0.136	0.155	0.215
**Specificity**	0.988	0.997	0.995	0.987	0.985	0.975
**PPV**	0.425	0.408	0.431	0.423	0.432	0.392
**NPV**	0.938	0.938	0.935	0.940	0.941	0.942
**F1**	0.182	0.062	0.097	0.206	0.228	0.278

Supplementary Table 7c. Binary classification performance (Low Use vs. High and Very High Use) for the 2023 overlap testing set.

	Logistic Regression	Support Vector Machine	Decision Tree	Random Forest	XGBoost	Multilayer Perceptron
**NA (%)**	2.39	8.99	3.44	3.40	2.80	0.00
**Accuracy**	0.844	0.859	0.841	0.851	0.850	0.837
**Balanced Accuracy**	0.625	0.558	0.572	0.639	0.642	0.652
**Sensitivity**	0.286	0.125	0.161	0.312	0.319	0.359
**Specificity**	0.965	0.992	0.983	0.965	0.964	0.945
**PPV**	0.637	0.734	0.672	0.655	0.655	0.597
**NPV**	0.862	0.862	0.848	0.869	0.868	0.867
**F1**	0.395	0.213	0.260	0.423	0.429	0.449

Supplementary Figure 1. F1 score curves across classification thresholds for each utilization class in the XGBoost model. The optimal thresholds were 0.46 for Low Use (blue), 0.17 for High Use (orange), and 0.20 for Very High Use (green), with a macro-averaged optimal threshold of 0.21 (black dashed line). These thresholds were selected to maximize classification performance for each class.

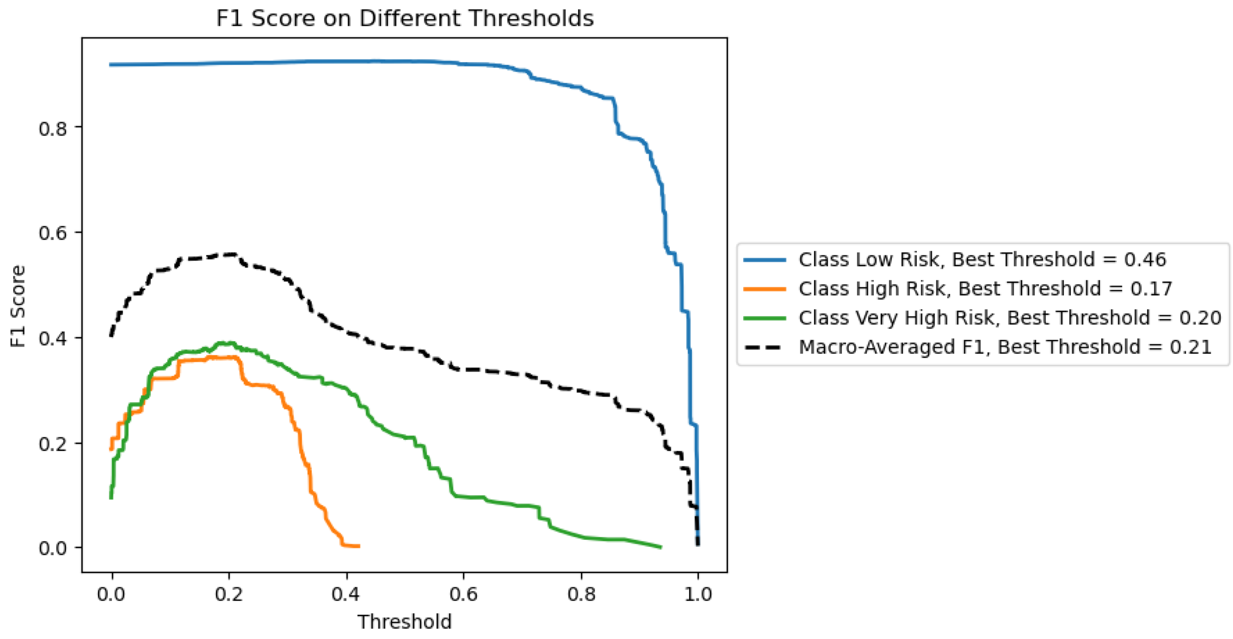

Supplementary Figure 2. SHAP analysis of the top predictive features in the XGBoost model for classifying primary care utilization groups. Bars represent the mean absolute SHAP value, indicating the average impact of each feature on the model's output magnitude. Colors correspond to utilization classes: Very High Use (red), High Use (orange), and Low Use (green).

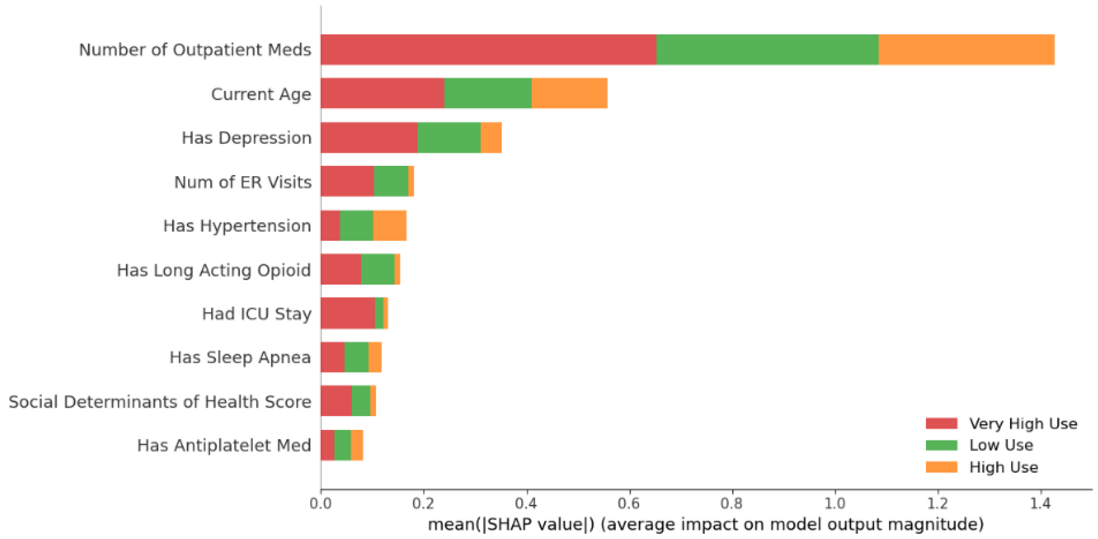

Supplementary Table 8. Study evaluation according to the PROBAST+AI guidelines.

	Domain	Quality (Model Development) / Risk of Bias (Model Evaluation)								Applicability
Model Development	Participants and Data Source	Data Source	Study Design	In- and Exclusion					Concern	Concern
		Y	PY	Y					Low	Low
	Predictor	Consistency	Preprocessing Uniformity	Blinded Assessment	Availability				Concern	Concern
		Y	Y	Y	PY				Low	Low
	Outcome	Appropriateness	Consistency	Blinded Assessment	Timing				Concern	Concern
		PY	Y	Y	Y				Low	Low
	Quality	Sample adequacy	Predictor Handling	Missing Data	Class Imablance	Overfitting			Concern	
		Y	Y	Y	NA				Low	
Model Evaluation	Participants and Data Source	Data Source	Study Design	In- and Exclusion					ROB	Concern
		Y	PY	Y					Low	Low
	Predictor	Consistency	Preprocessing Uniformity	Blinded Assessment	Availability				ROB	Concern
		Y	Y	Y	PY				Low	Low
	Outcome	Appropriateness	Consistency	Blinded Assessment	Timing				ROB	Concern
		PY	Y	Y	Y				Low	Low
	Analysis	Apparent Performance	Sample Adequacy	Missing Data	Class Imablance	Data Leakage	Resampling	Performance Evaluation	ROB	
		Y	Y	Y	NA	Y	NA	PY	Low	

Supplementary Table 9. Manuscript preparation according to the TRIPOD+AI checklist.

TRIPOD+AI

Section/Topic	Item	Development / evaluation[1]	Checklist item	Reported on page
**TITLE**				
Title	1	D;E	Identify the study as developing or evaluating the performance of a multivariable prediction model, the target population, and the outcome to be predicted	1
**ABSTRACT**				
Abstract	2	D;E	See TRIPOD+AI for Abstracts checklist	2
**INTRODUCTION**				
Background	3a	D;E	Explain the healthcare context (including whether diagnostic or prognostic) and rationale for developing or evaluating the prediction model, including references to existing models	3, 4
	3b	D;E	Describe the target population and the intended purpose of the prediction model in the context of the care pathway, including its intended users (e.g., healthcare professionals, patients, public)	4
	3c	D;E	Describe any known health inequalities between sociodemographic groups	
Objectives	4	D;E	Specify the study objectives, including whether the study describes the development or validation of a prediction model (or both)	4
**METHODS**				
Data	5a	D;E	Describe the sources of data separately for the development and evaluation datasets (e.g., randomised trial, cohort, routine care or registry data), the rationale for using these data, and representativeness of the data	7
	5b	D;E	Specify the dates of the collected participant data, including start and end of participant accrual; and, if applicable, end of follow-up	7
Participants	6a	D;E	Specify key elements of the study setting (e.g., primary care, secondary care, general population) including the number and location of centres	7
	6b	D;E	Describe the eligibility criteria for study participants	7
	6c	D;E	Give details of any treatments received, and how they were handled during model development or evaluation, if relevant	NA
Data preparation	7	D;E	Describe any data pre-processing and quality checking, including whether this was similar across relevant sociodemographic groups	10
Outcome	8a	D;E	Clearly define the outcome that is being predicted and the time horizon, including how and when assessed, the rationale for choosing this outcome, and whether the method of outcome assessment is consistent across sociodemographic groups	7
	8b	D;E	If outcome assessment requires subjective interpretation, describe the qualifications and demographic characteristics of the outcome assessors	NA
	8c	D;E	Report any actions to blind assessment of the outcome to be predicted	NA
Predictors	9a	D	Describe the choice of initial predictors (e.g., literature, previous models, all available predictors) and any pre-selection of predictors before model building	9
	9b	D;E	Clearly define all predictors, including how and when they were measured (and any actions to blind assessment of predictors for the outcome and other predictors)	9
	9c	D;E	If predictor measurement requires subjective interpretation, describe the qualifications and demographic characteristics of the predictor assessors	NA
Sample size	10	D;E	Explain how the study size was arrived at (separately for development and evaluation), and justify that the study size was sufficient to answer the research question. Include details of any sample size calculation	7
Missing data	11	D;E	Describe how missing data were handled. Provide reasons for omitting any data	10
Analytical methods	12a	D	Describe how the data were used (e.g., for development and evaluation of model performance) in the analysis, including whether the data were partitioned, considering any sample size requirements	12
	12b	D	Depending on the type of model, describe how predictors were handled in the analyses (functional form, rescaling, transformation, or any standardisation).	10, 12
	12c	D	Specify the type of model, rationale[2], all model-building steps, including any hyperparameter tuning, and method for internal validation	12
	12d	D;E	Describe if and how any heterogeneity in estimates of model parameter values and model performance was handled and quantified across clusters (e.g., hospitals, countries). See TRIPOD-Cluster for additional considerations[3]	NA
	12e	D;E	Specify all measures and plots used (and their rationale) to evaluate model performance (e.g., discrimination, calibration, clinical utility) and, if relevant, to compare multiple models	12,13
	12f	E	Describe any model updating (e.g., recalibration) arising from the model evaluation, either overall or for particular sociodemographic groups or settings	NA
	12g	E	For model evaluation, describe how the model predictions were calculated (e.g., formula, code, object, application programming interface)	13
Class imbalance	13	D;E	If class imbalance methods were used, state why and how this was done, and any subsequent methods to recalibrate the model or the model predictions	NA
Fairness	14	D;E	Describe any approaches that were used to address model fairness and their rationale	NA
Model output	15	D	Specify the output of the prediction model (e.g., probabilities, classification). Provide details and rationale for any classification and how the thresholds were identified	13

[1] D=items relevant only to the development of a prediction model; E=items relating solely to the evaluation of a prediction model; D;E=items applicable to both the development and evaluation of a prediction model

[2] Separately for all model building approaches.

[3] TRIPOD-Cluster is a checklist of reporting recommendations for studies developing or validating models that explicitly account for clustering or explore heterogeneity in model performance (eg, at different hospitals or centres). Debray et al, BMJ 2023; 380: e071018 [DOI: 10.1136/bmj-2022-071018]

Training versus evaluation	16	D;E	Identify any differences between the development and evaluation data in healthcare setting, eligibility criteria, outcome, and predictors	12
Ethical approval	17	D;E	Name the institutional research board or ethics committee that approved the study and describe the participant-informed consent or the ethics committee waiver of informed consent	6
**OPEN SCIENCE**				
Funding	18a	D;E	Give the source of funding and the role of the funders for the present study	27
Conflicts of interest	18b	D;E	Declare any conflicts of interest and financial disclosures for all authors	27
Protocol	18c	D;E	Indicate where the study protocol can be accessed or state that a protocol was not prepared	NA
Registration	18d	D;E	Provide registration information for the study, including register name and registration number, or state that the study was not registered	NA
Data sharing	18e	D;E	Provide details of the availability of the study data	28
Code sharing	18f	D;E	Provide details of the availability of the analytical code[4]	28
**PATIENT & PUBLIC INVOLVEMENT**				
Patient & Public Involvement	19	D;E	Provide details of any patient and public involvement during the design, conduct, reporting, interpretation, or dissemination of the study or state no involvement.	NA
**RESULTS**				
Participants	20a	D;E	Describe the flow of participants through the study, including the number of participants with and without the outcome and, if applicable, a summary of the follow-up time. A diagram may be helpful.	14
	20b	D;E	Report the characteristics overall and, where applicable, for each data source or setting, including the key dates, key predictors (including demographics), treatments received, sample size, number of outcome events, follow-up time, and amount of missing data. A table may be helpful. Report any differences across key demographic groups.	15, 16
	20c	E	For model evaluation, show a comparison with the development data of the distribution of important predictors (demographics, predictors, and outcome).	NA
Model development	21	D;E	Specify the number of participants and outcome events in each analysis (e.g., for model development, hyperparameter tuning, model evaluation)	14
Model specification	22	D	Provide details of the full prediction model (e.g., formula, code, object, application programming interface) to allow predictions in new individuals and to enable third-party evaluation and implementation, including any restrictions to access or re-use (e.g., freely available, proprietary)[5]	NA
Model performance	23a	D;E	Report model performance estimates with confidence intervals, including for any key subgroups (e.g., sociodemographic). Consider plots to aid presentation.	18-22
	23b	D;E	If examined, report results of any heterogeneity in model performance across clusters. See TRIPOD Cluster for additional details[3].	NA
Model updating	24	E	Report the results from any model updating, including the updated model and subsequent performance	NA
**DISCUSSION**				
Interpretation	25	D;E	Give an overall interpretation of the main results, including issues of fairness in the context of the objectives and previous studies	23
Limitations	26	D;E	Discuss any limitations of the study (such as a non-representative sample, sample size, overfitting, missing data) and their effects on any biases, statistical uncertainty, and generalizability	26
Usability of the model in the context of current care	27a	D	Describe how poor quality or unavailable input data (e.g., predictor values) should be assessed and handled when implementing the prediction model	NA
	27b	D	Specify whether users will be required to interact in the handling of the input data or use of the model, and what level of expertise is required of users	24
	27c	D;E	Discuss any next steps for future research, with a specific view to applicability and generalizability of the model	24-25

From: Collins GS, Moons KGM, Dhiman P, et al. *BMJ* 2024;385:e078378. doi:10.1136/bmj-2023-078378

[4] This relates to the analysis code, for example, any data cleaning, feature engineering, model building, evaluation.
[5] This relates to the code to implement the model to get estimates of risk for a new individual.
